# Churning the tides of care: when nurse turnover makes waves in patient access to primary care

**DOI:** 10.1186/s12912-024-02389-8

**Published:** 2024-10-10

**Authors:** Kelley Arredondo, Ashley M. Hughes, Houston F. Lester, Trang N.D. Pham, Laura A. Petersen, LeChauncy Woodard, Richard SoRelle, Cheng (Rebecca) Jiang, Frederick L. Oswald, Daniel R. Murphy, Hilary N. Touchett, Joshua Hamer, Sylvia J. Hysong

**Affiliations:** 1 Houston VA HSR&D Center for Innovations in Quality, Effectiveness, and Safety, Michael E DeBakey VA Medical Center, 2450 Holcombe Blvd Suite 01Y, Houston, TX 77021 USA; 2 Section of Health Services Research, Department of Medicine, Baylor College of Medicine, Houston, TX USA; 3 VHA Office of Rural Health’s Veterans Resource Center, White River Junction, USA; 4 South Central Mental Illness Research, Education, Clinical Center, a virtual center, Houston, USA; 5 Department of Medicine-MetroHealth, Case Western Reserve University School of Medicine, Cleveland, OH USA; 6 Center of Innovations for Chronic, Complex Healthcare, Edward Hines Jr VA Hospital, Hines, IL USA; 7 School of Business Administration, University of Mississippi, University, MS USA; 8 Department of Epidemiology and Biostatistics, School of Public Health, University of Illinois at Chicago, Chicago, IL USA; 9 Department of Psychological Sciences, Rice University, Houston, TX USA; 10 Michael E. DeBakey VA Medical Center, Houston, TX USA; 11 Psychiatry and Behavioral Sciences, Baylor College of Medicine, Houston, TX USA

**Keywords:** Registered nurses, RN, Staffing stability, Primary care teams, Patient aligned care teams, PACT

## Abstract

**Background:**

Team-based primary care (PC) enhances the quality of and access to health care. The Veterans Health Administration (VHA) implements team-based care through Patient Aligned Care Teams (PACTs), consisting of four core members: a primary care provider, registered nurse (RN) care manager, licensed vocational nurse, and scheduling clerk. RNs play a central role: they coordinate patient care, manage operational needs, and serve as a patient point of contact. Currently, it is not known how varying levels of RN staffing on primary care teams impact patient outcomes.

**Objective:**

This study aims to empirically assess how the stability of RN staffing within team-based primary care affects patient access to care.

**Methods:**

A retrospective database review using clinical and administrative data from the VHA over 24 months. Participants included 5,897 PC PACTs across 152 VHA healthcare facilities in the United States and its territories. The stability of personnel in the RN role was categorized as: RN continuous churn, RN staffing instability and RN vacancy. All 3 categories were compared to teams with RN stability (i.e., same person in the role for the entire 24-month period). Access measures included: average third-next-available appointment, established patient average wait time in days, urgent care utilization, emergency room utilization, and total inbound-to-outbound PC secure messages ratio.

**Results:**

RN continuous churn within PACTs had a significant impact on third-next-available appointment (b = 3.70, *p* < 0.01). However, RN staffing instability and vacancy had no significant relationship with any of the access measures. Several risk adjustment variables, including team full-time equivalency, team stability, relative team size, and average team size, were significantly associated with access to health care.

**Conclusions:**

Teams are impacted by churn on the team. Adequate staffing and team stability significantly predict patient access primary care services. Healthcare organizations should focus on personnel retention and strategies to mitigate the impact(s) of continuous RN turnover. Future research should examine the relative impact of turnover and stability of other roles (e.g., clerks) and how team members adapt to personnel changes.

Primary care uses a team-based approach that enhances quality and access to patient care, with nurses at its center [1–4]. As the largest health profession in the U.S., registered nurses (RNs) extend the capacity of primary care providers, and their inclusion in primary care teams improves patient care [5–8]. RNs serve as the care coordinator in many team-based primary care models, tasked with completing critical activities such as patient care coordination for complex and chronic disease management, patient education, patient messaging, and triage of clinic patients [1, 9, 10]. RNs are often tasked with managing daily operational needs, such as tracking patient health trends, and acting as a first point of contact for patient questions and follow-up needs [9, 10]. Therefore, RN shortages impact patient quality, efficiency, and can increase hospitals’ cost [10]. Despite the growing demand for primary care services, the primary care workforce - and the nursing workforce in particular -continues to decline [1, 11]. 

Furthermore, it is particularly difficult to staff and retain RNs in primary care due to various incentives attracting RNs to other clinical care settings (e.g., higher pay in hospitals, more flexible work schedules).Understandably, RN staffing shortages have direct and detrimental impacts to patient care activities. Two systematic reviews on the topic highlight how RN turnover, for instance, can yield greater burnout among retained nurses, which can subsequently impact quality and safety of patient care [12, 13]. RN churn, defined as a nurse frequently vacating their position on primary care teams [14–16], generates significant economic [1, 17–19] and non-economic (e.g., nursing well-being [20], patient-related outcomes [21–23]) costs, exacerbating known issues related to RN retention [24]. Specifically, RNs working in primary care are at high risk for turnover due to lower job satisfaction, higher burnout, and greater pay disparity than their counterparts working in hospital settings [1, 16, 19] all of which serve as strong predictors of RN churn [25–29]. RN churn coupled with RN shortages in primary care have hindered facilities’ ability to maintain stable RN staffing; clinics often resort to filling vacant RN primary care team roles with temporary RNs, float pool staff, part-time RNs, or re-assign RNs to fill RN responsibilities across multiple teams [20, 30]. These shortages were exacerbated during the COVID-19 pandemic where 22% of RNs reported the desire to leave their positions within a year due to the pandemic [31]. Yet, the impact of RN churn and instability in a team-based care setting (i.e., changes in who is filling the RN role at any given time in the primary care team) on patient access to care remains unknown.

This study aimed to address this knowledge gap directly by empirically testing the effect of the stability of RN role in team-based primary care on care quality measures of access. Given RNs’ pivotal role in primary care services, we hypothesized that the extent of RN churn on primary care teams would detrimentally impact patient access.

## Methods

### Design and participants

We conducted an integrated database review as part of a larger study [32] using clinical and administrative Veterans Health Administration (VHA) data sources. The database included 5,897 primary care Patient-Aligned Care Teams (PACTs) over 24 months (January 2019 to December 2020) that delivered care at 152 VHA health care facilities in the U. S. and its territories, including Veterans Affairs medical centers and community-based outpatient clinics.

### Setting

Within the VHA, team-based primary care is implemented nationwide in the form of PACTs, a VHA adaptation of the Patient-Centered Medical Home principles. PACTs consist of 4 core roles, which include a primary care provider, RN care manager, licensed vocational nurse, and scheduling clerk [33, 34]. Although the most common configuration is one full-time individual in each role, each role may be filled by multiple individuals at different full-time equivalent (FTE) portions to equal 1, thus increasing team size beyond 4. Each PACT is responsible for the care and coordination of a patient panel typically consisting of 1,200 patients [3]. 

### Data sources

Data were extracted from VHA’s Corporate Data Warehouse and Primary Care Almanac Team Assignments Report (TAR). Table 1 presents a summary of data sources with the corresponding variables extracted from each source and brief definitions for each variable.

**Table 1:** Data sources and definitions of study variables

Data Source	Data Source Description	Study variables	Definition
PACT Compass	Gives primary care managers and staff access to data on key metrics such as access, continuity of care and care coordination. The PACT Compass is updated nightly and created from fields within the Corporate Data Warehouse.	Average Third Next Available Appointment	The average waiting time in days between a completed appointment and the Third-Next-Available Appointment slot for each primary care clinic. A snapshot is taken on the first day of each month for the prior month’s activity. The wait times in days until the Third-Next-Available Appointment are averaged monthly for completed appointments.
		Established Primary Care Patient Average Wait Time in Days	The average number of calendar days between an established patient’s primary care completed appointment and earliest of 3 possible preferred (desired) dates from the completed appointment date.
		Urgent Care Utilization Rate	The total number of Urgent Care encounters for assigned primary care patients in the last 12 months divided by the team assignments.
		Emergency Room Utilization Rate	The total number of Emergency Room encounters for assigned primary care patients in the last 12 months divided by the team assignments.
		Inbound-to-outbound primary care secure messages ratio	A ratio representing the total number of secure messages sent by a patient assigned to a given primary care team divided by the total number of secure messages sent from a primary care team member to a patient assigned to that primary care team during the reporting period.
Team Assignments Report	Displays all active PACTs at every VAMC and CBOC within the VHA system, along with the names and roles of the primary care staff members assigned to each team. It is updated nightly and created from fields within the Corporate Data Warehouse, thereby facilitating linkage to other data sources for our study.	RN Stability	The same person in the RN role for the entire 24-month period.
		RN Continuous Churn	An RN role that remained filled throughout the 24 months, but by more than 1 individual.
		RN Staffing Instability	An RN role filled from 16.67–99% of the 24 months.
		RN Vacancy	An RN role filled less than 16.67% of the time.
		Full-Time Equivalent	The sum of each team member’s scheduled hours on a team divided by the number of hours for a full-time workweek.
		Team Stability	The team’s overall stability for the 24-month period, calculated as 1 minus the number of separations divided by the average number of team members where a score of 1 indicates that the entire team remained unchanged.
		Relative Team Size	The comparison of an increase in team size (i.e., number of people assigned to a team) at a given point relative to the team’s average size.
		Average Team Size	The average size of the team across the 24 months.

Note. PACT = patient-aligned care team; VAMC = Veterans Affairs medical center; CBOC = community-based outpatient clinic; VHA = Veterans Health Administration.

### Measures

**Main outcome: Access to primary care.** A recent study by Hysong and colleagues defined care quality within team-based primary care. The study identified 16 metrics to evaluate PACTs along three core objectives of primary care: access, partnership with patients, and technical quality [35]. Therefore, we used access measures as defined by Hysong and colleagues to determine RNs impact on access to care [35]. Access was operationalized using the following metrics: Average third-next-available appointment, established patient average wait time in days, urgent care (UC) utilization, emergency room (ER) utilization, and total inbound-to-outbound primary care secure messages ratio (number of messages received by PACTs divided by the number of messages *sent* by PACTs) [35–37]. 

#### Stability of personnel in RN role

**Fig. 1:**
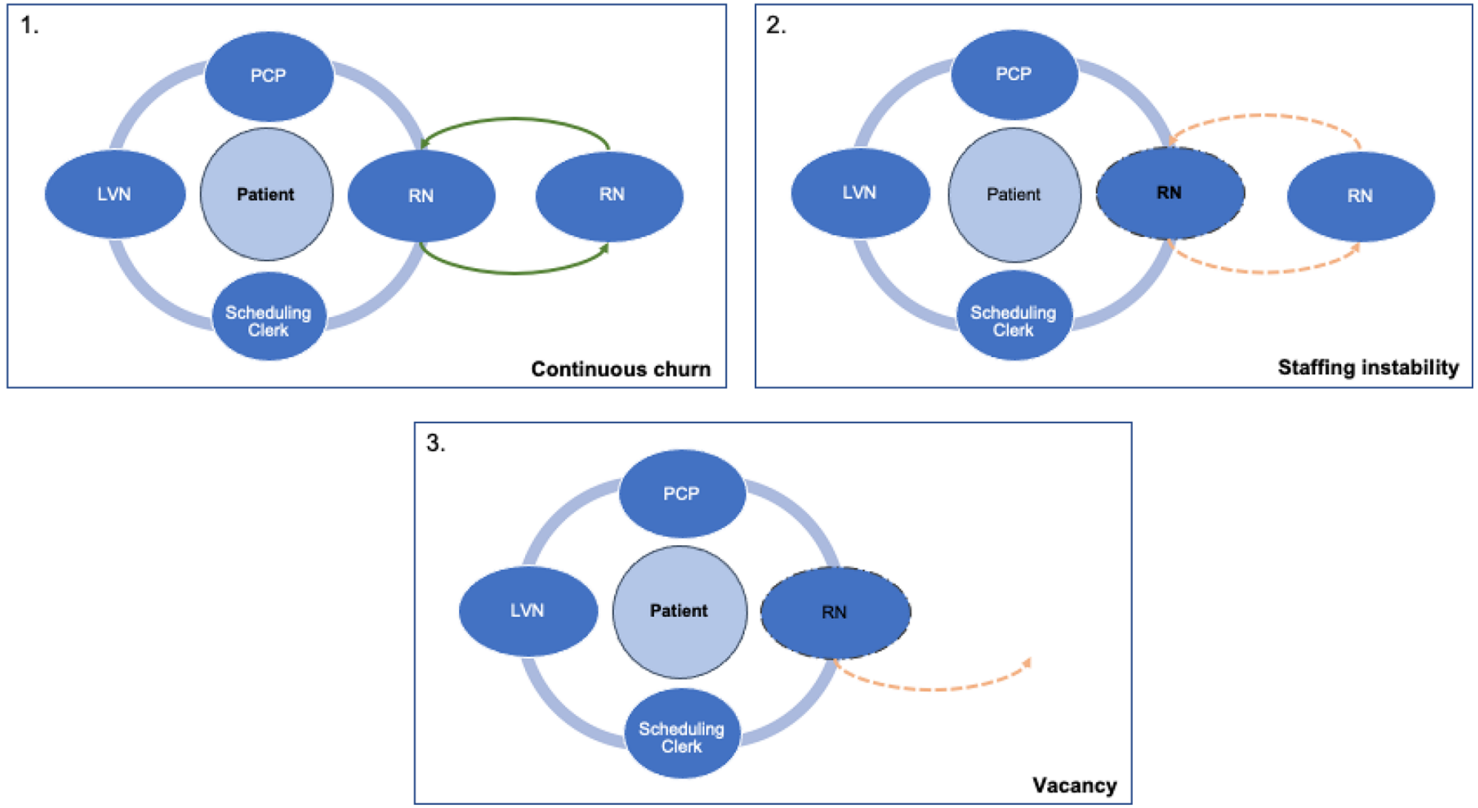
Depiction of three categories of stability of personnel in RN Role within primary care teams. Note. In panel 1, the green arrows with solid lines for the RN roles denotes continuous churn, where the position is always filled but by more than 1 individual. In panel 2, the orange dotted lines and dotted line around the RN role within the PACT denotes RN staffing instability, where the RN role is filled between 16.67 and 99% of the 24 months. In panel 3, the orange dotted arrow and dotted line around the PACT RN role denotes RN vacancy, where the RN role is filled less than 16.67% of the time during the 24 months

We calculated a multicategorical variable depicting the nature and extent to which RNs remained with their team during the observation period (see Fig. 1). Teams were classified as experiencing RN continuous churn if their RN role remained filled throughout the 24 months, but by more than 1 individual (Fig. 1, panel 1). RN staffing instability refers to teams that had their RN role filled from 16.67 to 99% of the 24 months (Fig. 1, panel 2), while RN vacancy encompasses teams whose RN role was filled less than 16.67% of the time (Fig. 1, panel 3). These cutoffs were determined by the natural breaks, given the distribution of the data. All 3 categories were compared to the reference category of RN stability, or teams with the same person in the RN role for the entire 24-month period.

#### Risk adjustments

To examine the impact of stability of personnel in the RN role, we risk-adjusted for the following variables known to influence team-based primary care: (a) team full-time equivalent (FTE), or the sum of each team member’s scheduled hours on a team divided by the number of hours for a full-time workweek; (b) team stability, defined as the team’s overall stability for the 24-month period, calculated as 1 minus the number of separations divided by the average number of team members, where a score of 1 indicates that the entire team remained unchanged; (c) average team size (level 2), which reflects the average size of the team across the 24 months; [33] and (d) relative team size, defined as the comparison of an increase in team size (i.e., number of people assigned to a team) at a given point relative to the team’s average size [38] (level 1), providing information on whether teams have more or fewer individuals than usual in a given month.

### Preliminary data analysis

**Fig. 2:**
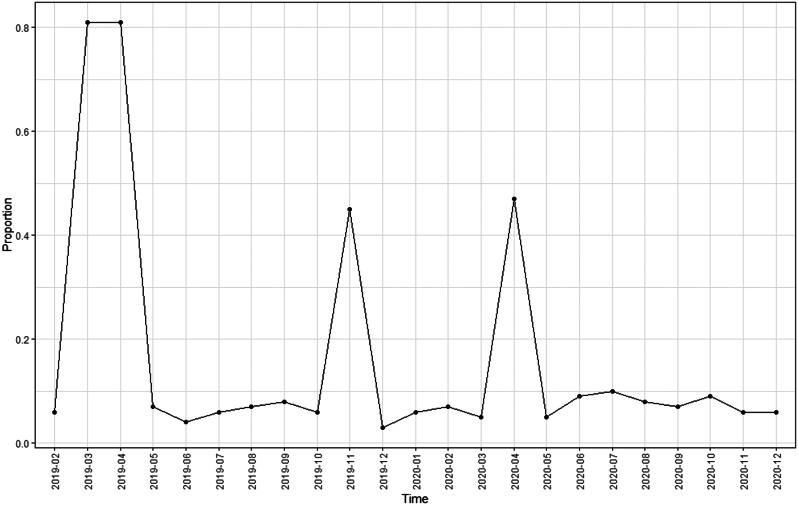
Proportion of PACTs with Nurse Turnover

To test for multicollinearity, we determined the variance inflation factor, which was always smaller than 3. This variance inflation factor is much smaller than the commonly used guideline of being less than ten [39]. Therefore, concerns of multicollinearity impacting analytic findings are minimal. Additionally, we determined the proportion of teams with nurse turnover across the 24 months (Fig. 2).

### Analytic strategy

To test our hypothesis, we conducted six random intercept multilevel models with each measure of access as the outcome (each access measure modeled separately). The random intercept allows us to account for the dependence due to time being nested within teams. Each outcome was predicted by the following level-two predictors: sum of team FTE, team size, overall team stability, RN continuous churn, RN staffing instability, and RN vacancy, with team stability as the only level-one predictor.

## Results

Table 2 provides the descriptive statistics of the access measures and Table 3 summarizes the characteristics of our sample. Table 4 provides a complete summary of our multilevel models including unstandardized regression coefficients.

RN staffing instability and vacancy on a team had no significant relationship with any of the access outcomes; however, we observed longer average third-next-available appointment times in teams experiencing RN continuous churn (b = 3.70, *p* < 0.01) compared to teams experiencing RN stability.

**Table 2:** Characteristics of VHA primary care team members and PACTs

Team-level Characteristics	*N*	%
**Clinical Focus**		
Primary Care only	5,738	61.41
Women’s Health	2,521	26.98
Academic	309	3.31
Geriatric Primary Care	275	2.94
Infectious Diseases	161	1.72
Spinal Cord Injury	157	1.68
Other	183	3.68
	***M***	***SD***
Team Size	3.75	0.72
Team FTE	2.57	1.31
Team Stability	-0.13	0.73

Note. *VHA = Veterans Health Administration; PACTS = patient-aligned care teams; FTE = fulltime equivalent*

**Table 3:** Descriptive statistics of access measures averaged across 24-month period

Outcome	M	SD
Average Third-Next-Available Appointment	20.70	27.54
Established PC Patient Wait Time in Days	3.89	7.88
UC Utilization	0.05	0.16
ER Utilization	44.32	37.37
Total Inbound to Total Outbound PC Secure Messages	1.65	3.12

Note. PC = primary care; UC = urgent care; ER = emergency room

Several risk factors were significantly associated with access. Of note, when risk-adjusting for all variables, greater team FTE was significantly related to better access across all measures. Further, team stability was associated with better access through average-third-next available appointment (b = -1.27, *p* < 0.01), established patient average wait time in days (b = -0.41, *p* < 0.001), and lower ER utilization (b = -4.10, *p* < 0.001). Relative team size was negatively related to inbound-to-outbound primary care secure messages ratio (b = -0.29, *p* < 0.001); however, relative team size was also associated with increased ER utilization (b = 0.45, *p* < 0.001) suggesting worse access to care. Lastly, greater average team size was associated with lower average third-next-available appointment times (b = -2.04, *p* < 0.001).

**Table 4:** Summary of parameter estimates (b) and standard errors resulting from multilevel model analyses

Outcome		RN Vacancy		RN Staffing Instability		RN Continuous Churn		Team Stability		Relative Team Size		Average team size		FTE
		No FTE	FTE	No FTE	FTE	No FTE	FTE	No FTE	FTE	No FTE	FTE	No FTE	FTE	FTE
Average Third Next Available Appointment	b	-2.41	-2.53	-0.68	-0.71	***3.94***	***3.70***	**-1.35**	**-1.27**	-0.26	-0.11	**-2.22**	**-2.04**	**-0.36**
	SE	2.38	2.38	0.71	0.71	***1.24***	***1.24***	**0.42**	**0.42**	0.15	0.17	**0.54**	**0.55**	**0.16**
Established PC Patient Wait Time in Days	b	-1.82	-1.98	-0.17	-0.21	-0.12	-0.50	**-0.50**	**-0.41**	-0.08	-0.09	**-0.50**	-0.21	**-0.55**
	SE	1.40	1.39	0.18	0.18	0.27	0.27	**0.10**	**0.10**	0.07	0.07	**0.14**	0.14	**0.06**
UC Utilization	b	-0.01	-0.01	0.01	0.01	-0.02	-0.02	0.001	0.001	***0.001***	0.0005	0.01	0.01	**0.0008**
	SE	0.02	0.02	0.007	0.01	0.02	0.02	0.004	0.004	***0.0003***	0.0003	0.005	0.005	**0.0004**
ER Utilization	b	1.18	0.67	-0.38	-0.52	-4.23	-4.63	**-4.25**	**-4.10**	0.05	***0.45***	-1.33	-1.00	**-0.88**
	SE	3.68	3.66	1.67	1.66	3.57	3.55	**1.02**	**1.01**	0.08	***0.10***	1.25	1.24	**0.11**
Total Inbound to Total Outbound PC Secure Messages	b	2.05	2.03	0.32	0.31	0.09	0.07	**-0.05**	-0.04	-0.31	**-0.29**	-0.09	-0.08	**-0.05**
	SE	0.24	0.24	0.06	0.06	0.13	0.13	**0.04**	0.04	0.03	**0.03**	0.05	0.05	**0.02**

Note Numbers in **bold type** indicate statistically significant differences in the clinically desired direction (e.g., lower ER utilization is better). Numbers in **bold italics** indicate statistically significant differences in the clinically undesirable direction (e.g., higher ER/UC utilization is worse). FTE = fulltime equivalent; PC = primary care; UC = urgent care; ER – emergency room

## Discussion

Our study leveraged one of the largest national samples of primary care teams to test the impact of RN churn in the team-based primary care setting on patient access to care, a high-priority objective in primary care settings [35]. We are not aware of other studies of comparable size that empirically examine the role of RN churn within the team-based primary care setting on evidence-driven measures of access [35, 40]. With this in mind, our study is positioned to extend understanding of RN churn as our results bear several implications for future practice and research.

First, we found that RN continuous churn hinders patient access to primary care by having longer wait time in days for average third-next-available appointment. Interestingly, we also found that neither RN staffing instability nor RN vacancy predicted any of the access outcomes. This does not mean that health care access was good, let alone that health care quality was not impacted, rather that access remained the same. Nevertheless, teams with RN staffing instability or RN vacancy had insufficient staffing, possibly due to barriers in recruiting new RNs or having very few PACTs making it nearly impossible to reassign RNs from one PACT to another [23, 41]. Over time, this additional workload can lead to burnout across all clinical team members [42, 43]. Conversely, larger clinics and those with proper resources can hire or reassign RNs across PACTs and maintain the RN role filled, which allows better workload distribution.

Secondly, the frequency of RN churn within a team has a differential impact on predicting meaningful differences in patient access. For instance, teams that experienced continuous churn within an RN role had longer average third-next-available-appointment times than teams that could not fill a vacant RN position. Part of the reason for this may be that frequent changes in personnel may create additional disruptions to team workflow, leading the team to continuously adjust to onboard and work with a new team-member, taking time away from patient-facing work (e.g., visits). Additionally, turnover often begets turnover; [25–29] thus, RNs who are cycling in to fill the position in teams with continuous churn may be joining already dysfunctional teams -- for example, with low morale due to churn, that fail to support new team members, have poor leadership or working conditions -- which may perpetuate and/or explain the presence of continuous churn. Providing RNs with organizational support and better working conditions to promote job satisfaction can not only help RNs stay within a team but also improve patient care [44]. 

Additionally, our results highlight the importance of staffing for the whole team, not just the RN role. FTE for the team as a risk adjustment significantly predicted each measure of patient access. This finding signals the meaningful benefits associated with adequately and sufficiently staffing PACTs such that patients can use preventive care services in a timely fashion (i.e., prior to the development of an emergency). Conversely, the addition of a team member (i.e., relative team size) suggests mixed effects for access. Though maintaining a stable team is not always feasible in a practice setting, the finding suggests that having a stable team with sufficient FTE can positively influence patients’ access to care.

Taken together, our results demonstrate that teams may be impacted most, whether positively or negatively, when there is churn on the team. Specifically, RN continuous churn had significant effects on primary care access. For teams with RN staffing instability or RN vacancy, the other team members were constant, perhaps providing a form of team stability. This suggests team stability not only benefits teams with no churn but also allows teams with churn some time to adapt to the change. PACTs consist of interprofessional team members who may adjust to the removal of a team member by clarifying expectations and assigning or re-assigning tasks as a collective; thus, stable teams may enable compensatory behaviors [45](e.g., adjustments in tasking) when a critical team member, such as the RN, is removed. Nevertheless, although primary care teams may adapt to an unfilled RN position in the short-term, the overburdening of remaining staff can impose higher workload and lower job satisfaction, making the team increasingly vulnerable to clinician burnout [8]. In fact, previous studies have found that RNs report poor staffing and high workload as important contributors to intention to leave [24, 46]. 

### Implications

Overall, these findings highlight that continuous turnover in the RN role, team staffing, and team stability predict meaningful differences in patient access to primary care services. Our findings bear several practical implications for health care organizations seeking to make meaningful improvements in patient access. First, interventions could be deployed to target the factors influencing RN churn to promote team stability and mitigate the deleterious effects of continuous churn. For instance, policymakers could identify high priority RN retention incentives (e.g., ongoing professional development, competitive pay/job benefits, alternative work schedules) to mitigate the likelihood of continuous churn in RN roles. This could manifest as organizational policy to ensure pay equity for long-term employees or increasing benefits based on tenure with the company [47–49]. 

At the team level, our results suggest that although fully staffed and larger teams improve access to care, teams that add a new team member (i.e., relative team size) can cause disruptions that (at least temporarily) negatively impact access to care, such as increased ER utilization. This may be caused by the additional resources that the team as a whole has to exert to onboard a new team-member; it also highlights that it takes time to reap the benefits of having a new team-member, since it takes time and multiple interactions to develop the processes and conditions associated with effective team performance, such as team coordination and communication, mental models and transactive memory [50–52]. Consequently, teams with continuous churn, nearly always in onboarding mode, never reap the full benefits of the new team member. Therefore, hospitals and clinics should develop structured onboarding processes to help new team members assimilate to the organization and team and mitigate the additional workload a new member poses on team members [53]. Conversely, larger PACTs may have greater bandwidth for adjusting to the removal of a critical team member, such as an RN, and may be more robust to changes to team membership as a whole. We are not advocating for larger teams, as this may introduce other issues in team dynamics [54] and access; nevertheless, organizations should provide teams with tools and dedicated flex personnel who are familiar with the team to enable teams to adjust to changes in personnel (addition or removal of a team member), as these individuals are already acquainted with the team dynamics, enabling them to seamlessly step in when needed, preserving key indicators of care quality, such as access.

Overall, the implications of these results are significant and suggest that there are several factors that can be addressed to improve access to care in primary care teams. By implementing targeted strategies and policies, healthcare organizations and policymakers may improve RN retention, thereby enhancing team stability and improving access to care.

### Limitations

This study relies on data reported in administrative and clinical databases. Thus, it is limited, based on availability of the data. Of note, we used the access measures as described by Hysong and colleagues [35] and agree that a new patient average wait time in days is a measure of access; however, we did not include it in the current study. Data definitions for this variable in the VHA highlight that enrolling a new patient undergoes a standardized process by which new patients are assigned to PACTs by an external administrator rather than scheduled by a working member of the PACT (e.g., clerk). Therefore, this measure is outside the scope of this study, as this is outside PACTs control. Additionally, staffing data reported within the administrative database (TAR) was used to calculate RN turnover for the study; however, this database does not account for temporary absences of personnel due to extended leave (e.g., maternity). Further, if an RN exits and reenters the VHA system, the database treats this person as a new RN, meaning that the data source cannot account for RNs returning to the primary care setting in the VHA. Moreover, in the specific setting where this work was performed (i.e., VHA), loss of a primary care provider dissolves the PACT, meaning that turnover within the team stability metric (which was also calculated using the TAR) would have had to come from RNs or clerks. Although examining turnover by team role was beyond the scope of this study, further investigation is warranted to examine the influence and contribution of turnover in other key team member roles within PACTs outside nursing.

Furthermore, our study’s definition of ‘access’ is defined primarily in the form of clinic accessibility that includes patients’ ability to schedule appointments and communicate with members of the care team [55]. The measures of access used in this study are the only measures that are available nationwide and via electronic health record databases. However, these measures have their limitations. For instance, total inbound-to-outbound primary care secure messages ratio is only captured if messages are sent through the patient’s portal, but primary care providers frequently call the patient directly to address their concerns rather than asking the nurse to type out a response in the patient portal. Therefore, a lack of response to a message in the patient portal may not signify less access due to team workflow differences [56]. Other measures of patient access are in the form of survey data, which differ in terms of completeness, response rate, and type of survey by institution. We hope this will be a first step in identifying more direct and objective measures for the important concept of ‘access’, particularly in light of patient centered medical home stated goals. There are additional factors to consider when considering the concept of ‘access’ as a whole. For instance, the different geographic locations of each VHA healthcare facility can impact access where many rural areas are designated Health Professional Shortage Areas and rural residents have to travel further for their care compared to urban residents [57, 58]. 

## Conclusion

RNs play a significant role in patient access to team-based care; although RN churn can have harmful effects on patient access to preventive care services via primary care, FTE of a team is a consistent predictor across several measures of access. Therefore, health systems should work to ensure adequate staffing in team-based care models in terms of FTE and size even in the absence of a critical role (e.g., RN) to avoid lapses in patient access. With the increased attention on team-based care metrics [35, 59], future research should identify how different team-member roles my differentially impact primary care team performance and should seek to prepare and optimize team members’ ability to work with and adapt to fluctuations in roles and personnel.

## Data Availability

Per the terms of our data use agreements with data owners, identifiable data cannot be shared. A limited, de-identified data set may be obtained by written request to the corresponding author per VA policy.

## Abbreviations

ER: Emergency room
FTE: Full time equivalent
PACT: Patient aligned care teams
PC: Primary care
RN: Registered nurse
TAR: Team assignments report
VHA: Veterans’ health administration
